# Earlobe Creases as a Marker of the Risk for Coronary Atherosclerosis Before Angiography in Elderly and Non-elderly Patients

**DOI:** 10.7759/cureus.36609

**Published:** 2023-03-23

**Authors:** Osamu Sasaki, Toshihiko Nishioka, Hideki Sasaki

**Affiliations:** 1 Internal Medicine, Kouiki Mombetsu Hospital, Mombetsu, JPN; 2 Cardiology, Saitama Medical Center, Saitama Medical University, Kawagoe, JPN; 3 Cardiovascular Surgery, Nagoya City University East Medical Center, Nagoya, JPN

**Keywords:** earlobe crease, non-elderly patient, elderly patient, gensini score, coronary artery disease

## Abstract

Background

The close relationship between earlobe creases (ELC) and the presence of coronary artery disease (CAD) has been reported. In addition, this study aimed to determine associations between ELC and the presence, extent, and severity of coronary atherosclerosis assessed by coronary angiography in non-elderly and elderly patients.

Methods

We assessed 1,086 consecutive patients with suspected CAD by coronary angiography. We defined severe CAD as Gensini scores > 20. Multiple logistic regression analysis was adjusted for age, sex, hypertension, diabetes mellitus, smoking status, lipid profiles, and body mass index (BMI) to assess the presence or absence of CAD, multivessel disease, and severe CAD in elderly (age ≥ 60 years) and non-elderly (age < 60 years) patients.

Results

ELC was a significantly positive determinant of CAD (odds ratio (OR) = 3.074, p < 0.001), multivessel disease (OR = 3.101, p < 0.001), and severe CAD (OR = 2.823, p < 0.001) in all patients. ELC was also a predictor of CAD, multivessel disease, and severe CAD not only in patients aged ≥ 60 years (OR = 3.095, p < 0.001; OR = 3.071, p < 0.001; OR = 2.761, p < 0.001, respectively) but also in those aged < 60 years (OR = 2.749, p = 0.035; OR = 2.634, p = 0.038; OR = 2.766, p = 0.006, respectively).

Conclusions

ELC was independently associated with the presence of CAD, multivessel disease, and severe CAD in both elderly and non-elderly patients who were assessed by coronary angiography.

## Introduction

Frank (1973) [1] initially described the relationship between earlobe creases (ELC) and coronary artery disease (CAD). Some subsequent studies found a significant positive relationship between ELC and CAD [2,3], whereas others did not [4,5]; thus, conclusions have remained controversial. Moreover, a relationship between the angiographic severity of CAD and ELC has been implied [6,7] but not confirmed, and the increasing prevalence of ELC with aging and their clinical significance according to age is unclear.

The present study aimed to clarify the association between ELC and the presence, extent, and severity of CAD in non-elderly and elderly patients who were assessed by coronary angiography.

## Materials and methods

Participants

This study included a series of 1,086 consecutive patients (mean age: 66.1 ± 11.4 years; males: 826) who were assessed by coronary angiography at Saitama Medical Center, Saitama Medical University, located in Kawagoe City, Saitama Prefecture, Japan, between February 2009 and September 2012. The exclusion criteria comprised a history of coronary artery bypass grafts and percutaneous coronary intervention (PCI). Our institutional review board at Saitama Medical Center, Saitama Medical University (approval number: 1116) approved the study, and all patients provided written informed consent to participate.

The patients were divided into groups according to the presence or absence of ELC. The severity of coronary atherosclerosis was assessed using Gensini scores [8], and coronary risk factors such as age, sex, hypertension, dyslipidemia, diabetes mellitus, and smoking were identified from a review of the medical records of the patients. The effects of angiotensin-converting enzyme inhibitors/angiotensin II receptor blockers (ACEI/ARB) and statins, as well as body mass index (BMI) and low-density lipoprotein cholesterol (LDL-C) and high-density lipoprotein cholesterol (HDL-C) levels, were also assessed.

Definitions

Body mass index was calculated as weight (kg) divided by height squared (m^2^). Coronary artery disease was defined as >50% luminal narrowing of any main coronary artery on coronary angiograms. Multivessel disease was defined as >50% luminal narrowing in at least two of the left anterior descending, left circumflex, and right coronary arteries. Severe CAD was defined as Gensini scores ≥ 20 [9]. Dyslipidemia was defined as values of >220, >140, <40, and >150 mg/dL for total cholesterol, LDL-C, HDL-C, and triglycerides, respectively, or taking lipid-lowering medication. Smoking was defined as currently smoking or having stopped smoking <1 month before enrollment. Hypertension was defined as systolic or diastolic blood pressure > 140 and/or > 90 mmHg, respectively, or under medication with antihypertensive drugs. Diabetes was defined as a confirmed diagnosis of diabetes mellitus or under antidiabetic medication with insulin or oral hypoglycemic agents at enrollment.

Coronary angiography

We evaluated the severity of CAD using Gensini scores [8]. Severe CAD was defined as Gensini scores ≥ 20, which was equivalent to 90% stenosis at a proximal site in the left anterior descending coronary artery [9]. The patients were divided according to Gensini scores of ≥20 (severe CAD) and <20 (mild CAD).

ELC classification

**Figure 1:**
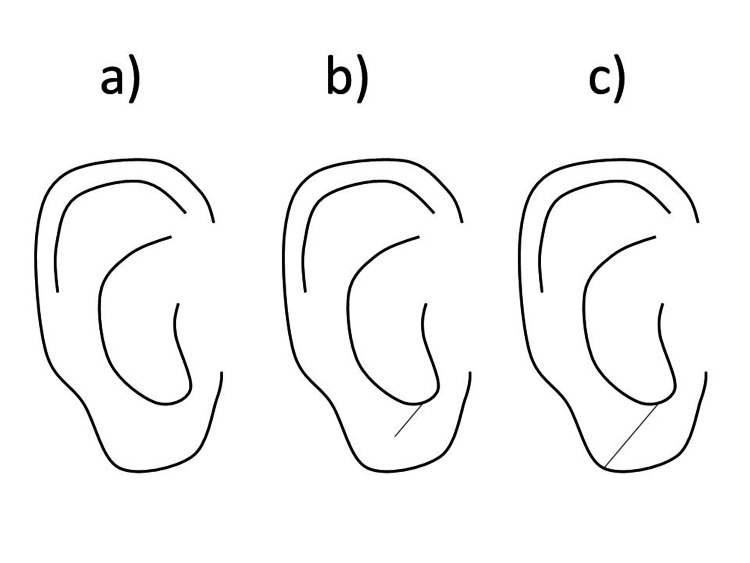
Earlobe crease a, b, and c show representative schema demonstrating grades 0, 1, and 2, respectively.

Earlobe creases were generally assessed in patients while seated or supine if they were in an unstable clinical condition such as shock. Two trained observers who were blinded to the coronary angiographic findings graded ELC as absent (grade 0), superficial or not completely extending across the earlobe (grade 1), or clear-cut creases extending across the earlobe (grade 2) [10]. Figure 1 illustrates a schema that outlines the grading of earlobe creases.

Statistics

All data were analyzed using the Statistical Package for the Social Sciences (SPSS) software version 22.0 (IBM SPSS Statistics, Armonk, NY, USA). Continuous variables are expressed as mean ± standard deviation (SD), and categorical variables are expressed as numbers (n) with frequency (%). Continuous and categorical variables were compared using unpaired t-tests and chi-square tests, respectively. Associations between ELC and coronary atherosclerosis such as CAD, multivessel disease, and severe CAD were assessed using multiple logistic regression analyses. Odds ratios (ORs) and 95% confidence intervals (CIs) for coronary atherosclerosis were calculated from the outcomes of multiple logistic regression models after adjustment for the potential confounders of age, sex, hypertension, diabetes mellitus, smoking status, LDL-C, HDL-C, and BMI.

## Results

Clinical characteristics and angiographic findings of patients with and without ELC

Table 1 shows the baseline characteristics of the patients. We found that 841 (77.4%) of them had ELC and 245 (22.5%) did not. The frequencies of gender, dyslipidemia, smoking, and plasma HDL-C values did not significantly differ between the two groups. However, patients with ELC were older (68.6 ± 9.3 versus 58.1 ± 13.2, p < 0.001) and had lower plasma LDL-C values (111.6 ± 34.7 versus 118.4 ± 33.2, p = 0.008) than those without ELC.

**Table 1:** Characteristics of the patients Data are shown as mean ± SD or number (%). SD, standard deviation; ELC, earlobe crease; ACE-I, angiotensin-converting enzyme inhibitor; ARB, angiotensin II receptor blocker; LDL-C, low-density lipoprotein cholesterol; HDL-C, high-density lipoprotein cholesterol; BMI, body mass index

	All (n = 1086)	With ELC (n = 841)	Without ELC (n = 245)	p value
Age (years)	66.1 ± 11.4	68.6 ± 9.3	58.1 ± 13.2	<0.001
Male	826 (76.1%)	650 (77.3%)	176 (71.7%)	0.078
Hypertension	771 (70.9%)	626 (74.4%)	145 (59.2%)	<0.001
Diabetes mellitus	387 (35.6%)	313 (37.2%)	74 (30.2%)	0.048
Dyslipidemia	681 (62.7%)	536 (63.7%)	145 (59.2%)	0.194
Smoking	419 (38.6%)	316 (37.6%)	103 (41.9%)	0.206
ACE-I/ARB	362 (33.3%)	296 (35.2%)	66 (26.9%)	0.015
Statins	264 (24.3%)	218 (25.9%)	46 (18.7%)	0.021
LDL-C (mg/dL)	113.2 ± 34.4	111.6 ± 34.7	118.4 ± 33.2	0.008
HDL-C (mg/dL)	45.8 ± 12.8	45.6 ± 12.5	46.5 ± 13.5	0.367
BMI (kg/m^2^)	23.9 ± 3.5	23.6 ± 3.1	24.6 ± 4.1	<0.001

The frequencies of hypertension, diabetes mellitus, and medication with ACE-I/ARB and statins were significantly higher in patients with ELC than those without (74.4% versus 59.2%, p < 0.001; 37.2% versus 30.2%, p = 0.048; 35.2% versus 26.9%, p = 0.015; 25.9% versus 18.7%, p = 0.021, respectively).

The frequencies of CAD and multivessel disease were higher (88.3% versus 68.9%, p < 0.001; 34.5% versus 21.2%, p < 0.001, respectively) and the sum of Gensini scores was significantly higher in patients with ELC than those without (46.7 ± 37.6 versus 31.9 ± 37.2, p < 0.001) (Table 2). No significant differences were evident between unilateral and bilateral ELC, or between ELC grades 1 and 2.

**Table 2:** Angiographic findings ELC, earlobe crease; CAD, coronary artery disease; VD, vessel disease

	All (n = 1086)	With ELC (n = 841)	Without ELC (n = 245)	p value
CAD (%)	912 (83.9%)	743 (88.3%)	169 (68.9%)	<0.001
0VD (%)	201 (18.5%)	115 (13.7%)	86 (35.2%)	<0.001
1VD (%)	543 (50%)	436 (51.8%)	107 (43.8%)	0.024
2VD (%)	227 (20.9%)	193 (23%)	34 (13.7%)	0.002
3VD (%)	115 (10.6%)	97 (11.5%)	18 (7.3%)	0.061
Multivessel disease (%)	342 (31.5%)	290 (34.5%)	52 (21.2%)	<0.001
Gensini score	41.5 ± 38.0	46.7 ± 37.6	31.9 ± 37.2	<0.001

Associations between ELC and CAD, multivessel disease, and severe CAD in all patients

Multiple logistic regression analysis showed that ELC and the classical coronary risk factors male sex, hypertension, smoking, high LDL-C, and low HDL-C were independent risk factors for coronary atherosclerosis. The presence of ELC was a predictor of the presence of CAD (OR = 3.074, p < 0.001), multivessel disease (OR = 3.101, p < 0.001), and severe CAD (OR = 2.823, p < 0.001) in all patients (Tables 3-5). The OR of ELC was the highest among all risk factors for CAD.

**Table 3:** Multiple logistic regression analysis of the presence of CAD in all patients CAD, coronary artery disease; OR, odds ratio; CI, confidence interval; LDL-C, low-density lipoprotein cholesterol; HDL-C, high-density lipoprotein cholesterol; BMI, body mass index; ELC, earlobe crease

	OR	95% CI	p value
Age	1.021	0.999-1.044	0.052
Male	2.103	1.279-3.455	0.003
Hypertension	2.543	1.615-4.002	<0.001
Diabetes mellitus	1.362	0.824-2.252	0.227
Smoking	1.963	1.188-3.244	0.008
LDL-C	1.010	1.003-1.017	0.002
HDL-C	0.970	0.955-0.986	<0.001
BMI	0.989	0.929-1.053	0.748
ELC	3.074	1.889-5.002	<0.001

**Table 4:** Multiple logistic regression analysis of multivessel disease in all patients OR, odds ratio; CI, confidence interval; LDL-C, low-density lipoprotein cholesterol; HDL-C, high-density lipoprotein cholesterol; BMI, body mass index; ELC, earlobe crease

	OR	95% CI	p value
Age	1.018	0.997-1.040	0.084
Male	2.131	1.318-3.447	0.002
Hypertension	2.395	1.540-3.722	<0.001
Diabetes mellitus	1.242	0.773-1.995	0.369
Smoking	2.085	1.287-3.376	0.002
LDL-C	1.011	1.005-1.018	<0.001
HDL-C	0.971	0.956-0.986	<0.001
BMI	0.965	0.909-1.025	0.259
ELC	3.101	1.938-4.962	<0.001

**Table 5:** Multiple logistic regression analysis of CAD severity in all patients CAD, coronary artery disease; OR, odds ratio; CI, confidence interval; LDL-C, low-density lipoprotein cholesterol; HDL-C, high-density lipoprotein cholesterol; BMI, body mass index; ELC, earlobe crease

	OR	95% CI	p value
Age	1.001	0.984-1.018	0.884
Male	1.787	1.195-2.670	0.004
Hypertension	1.991	1.383-2.868	<0.001
Diabetes mellitus	1.097	0.764-1.575	0.613
Smoking	1.256	0.870-1.814	0.222
LDL-C	1.005	1.000-1.010	0.041
HDL-C	0.966	0.954-0.979	<0.001
BMI	0.967	0.919-1.017	0.202
ELC	2.823	1.905-4.184	<0.001

Associations between the presence of ELC and the presence of CAD, multivessel disease, and severe CAD in patients aged ≥60 and <60 years

The presence of ELC was a predictor of the presence of CAD, multivessel disease, and severe CAD in patients aged ≥60 years (OR = 3.095, p < 0.001; OR = 3.071, p < 0.001; OR = 2.761, p < 0.001, respectively) and in those aged <60 years (OR = 2.749, p = 0.035; OR = 2.634, p = 0.038; OR = 2.766, p = 0.006, respectively) (Tables 6, 7).

**Table 6:** Associations between ELC and the presence of CAD in patients aged ≥60 years Adjusted for age, sex, hypertension, diabetes mellitus, smoking status, low-density lipoprotein cholesterol, high-density lipoprotein cholesterol, and body mass index ELC, earlobe crease; CAD, coronary artery disease; OR, odds ratio; CI, confidence interval

	OR	95% CI	p value
Presence of CAD	3.095	1.731-5.533	<0.001
Multivessel disease	3.071	1.751-5.384	<0.001
Severity of CAD	2.761	1.703-4.477	<0.001

**Table 7:** Associations between ELC and the presence of CAD in patients aged <60 years Adjusted for age, sex, hypertension, diabetes mellitus, smoking status, low-density lipoprotein cholesterol, high-density lipoprotein cholesterol, and body mass index ELC, earlobe crease; CAD, coronary artery disease; OR, odds ratio; CI, confidence interval

	OR	95% CI	p value
Presence of CAD	2.749	1.072-7.049	0.035
Multivessel disease	2.634	1.053-6.588	0.038
Severity of CAD	2.766	1.331-5.751	0.006

## Discussion

We identified two important clinical issues. The presence of ELC was independently associated with the presence of CAD, multivessel disease, and severe CAD assessed by coronary angiography. The presence of ELC was a more powerful indicator of CAD than conventional coronary risk factors in both elderly patients and non-elderly patients who were assessed by coronary angiography.

Earlobe creases and the severity of coronary atherosclerosis

An association between ELC and coronary atherosclerosis has been identified in clinical [1-3,11-13] and necropsy [14-16] studies. A previous meta-analysis [12] has revealed a potential association between ELC and the presence of coronary artery disease, with an odds ratio of 3.3. Earlobe creases are also significantly related to atherosclerotic changes in the carotid artery [17] and the aorta [14]. Elliott et al. [6] followed up on patients admitted to a coronary care unit or who underwent cardiac catheterization and found an association between the number of creased ears and 10-year cardiac events.

The association between ELC and atherosclerosis was evaluated using various methods, including carotid ultrasonography [17], CT angiography [18], coronary angiography [9], and autopsy or necropsy [14-16]. To the best of our knowledge, only one study has identified an association between ELC and the severity of coronary atherosclerosis using CT angiography [18], and the present study is the first to demonstrate a relationship between ELC and the severity of CAD based on a semiquantitative scoring system for coronary angiography.

Clinical implications for elderly and non-elderly populations

The present findings identified ELC as the most powerful indicator of coronary atherosclerosis among elderly and non-elderly patients and that age, diabetes mellitus, and BMI (or obesity) were not significantly associated with coronary atherosclerosis in this population. Aging is one of the most important risk factors for many diseases, and it is generally considered to be significantly associated with atherosclerosis. Indeed, previous studies [3,19,20] have shown a relationship between age and CAD. However, one autopsy study [14] found no association between age and the extent of coronary aortic atherosclerosis, whereas ELC was significantly associated with both coronary and aortic atherosclerosis. Age was no longer considered a predictor of CAD in that population, whereas ELC remained a significant indicator of CAD.

The present study of a high-risk population with suspected CAD did not find any association between age and CAD but identified ELC as the most significant determinant of coronary atherosclerosis in both elderly and non-elderly populations. This finding is consistent with the outcomes of a previous meta-analysis [12]. Therefore, ELC could be a useful marker for the risk stratification of patients before coronary angiography regardless of age.

Mechanism and future

One explanation for an association between the myocardium and earlobe has been that both are supplied by the same end arteries, of which the origin is genetically determined. Shoenfeld et al. [21] found significant tears in the elastic fibers of biopsy specimens of earlobes from patients with ELC, and Higuchi et al. [10] found that patients with ELC had shorter telomeres. However, the precise mechanism of the association between ELC and CAD has not been clearly elucidated. Further investigation is needed to clarify the mechanism of this relationship.

Limitations

As a limitation of this study, it is important to note that it is a single-center, cross-sectional, and observational investigation, which differs significantly from a randomized controlled trial. The study included high-risk patients who underwent coronary angiography. In fact, 83.9% of the patients had significant CAD defined as >50% stenosis on coronary angiograms. Therefore, the present findings cannot be expanded to healthy or asymptomatic individuals. Race, family history of coronary artery disease, and other possible confounding factors were important variables but were not included in our study.

## Conclusions

Earlobe creases were independently associated with the presence of CAD, multivessel disease, and severe CAD in elderly and non-elderly patients who were assessed by coronary angiography. Earlobe creases could serve as a useful marker for the risk stratification of patients before undergoing coronary angiography regardless of age.
